# Branched-chain amino acids and muscle protein synthesis in humans: myth or reality?

**DOI:** 10.1186/s12970-017-0184-9

**Published:** 2017-08-22

**Authors:** Robert R. Wolfe

**Affiliations:** University of Arkansas for Medical Sciences, 4301 West Markham street, slot 806, Little Rock, AR 72205-7199 USA

**Keywords:** Leucine, Valine, Isoleucine, Humans, Anabolic response

## Abstract

The branched chain amino acids (BCAAs) are leucine, valine and isoleucine. A multi-million dollar industry of nutritional supplements has grown around the concept that dietary supplements of BCAAs alone produce an anabolic response in humans driven by a stimulation of muscle protein synthesis. In this brief review the theoretical and empirical bases for that claim are discussed. Theoretically, the maximal stimulation of muscle protein synthesis in the post-absorptive state in response to BCAAs alone is the difference between muscle protein breakdown and muscle protein synthesis (about 30% greater than synthesis), because the other EAAs required for synthesis of new protein can only be derived from muscle protein breakdown. Realistically, a maximal increase in muscle protein synthesis of 30% is an over-estimate because the obligatory oxidation of EAAs can never be completely suppressed. An extensive search of the literature has revealed no studies in human subjects in which the response of muscle protein synthesis to orally-ingested BCAAs alone was quantified, and only two studies in which the effect of intravenously infused BCAAs alone was assessed. Both of these intravenous infusion studies found that BCAAs decreased muscle protein synthesis as well as protein breakdown, meaning a decrease in muscle protein turnover. The catabolic state in which the rate of muscle protein breakdown exceeded the rate of muscle protein synthesis persisted during BCAA infusion. We conclude that the claim that consumption of dietary BCAAs stimulates muscle protein synthesis or produces an anabolic response in human subjects is unwarranted.

## Background

There are a total of twenty amino acids that comprise muscle protein. Nine of the twenty are considered essential amino acids (EAAs), meaning they cannot be produced by the body in physiologically significant amounts, and therefore are crucial components of a balanced diet. Muscle protein is in a constant state of turnover, meaning that protein synthesis is occurring continuously to replace protein lost as a consequence of protein breakdown. For synthesis of new muscle protein, all the EAAs, along with the eleven non-essential amino acids (NEAAs) that can be produced in the body, must be present in adequate amounts. The branched-chain amino acids leucine, isoleucine and valine are three of the nine EAAs. Leucine is not only a precursor for muscle protein synthesis, but also may play a role as a regulator of intracellular signaling pathways that are involved in the process of protein synthesis (e.g., [1]).

The concept that the BCAAs may have a unique capacity to stimulate muscle protein synthesis has been put forward for more than 35 years. Data supporting this hypothesis have been obtained from studies of the responses of rats. In 1981 Buse [2] reported that in rats the BCAAs may be rate limiting for muscle protein synthesis. Additional studies supported the concept of a unique effect of BCAAs on muscle protein synthesis in rats, although few have studied the response to oral consumption of only BCAAs. Garlick and Grant showed that infusion of a mixture of BCAAs into rats increased the rate of muscle protein synthesis in response to insulin [3], but they did not measure the effects of BCAAs alone. The infusion of BCAAs alone into rats by Kobayashi et al. [4] was shown to induce an increase in muscle protein synthesis, but the response was only transient. Presumably the rate of synthesis quickly became limited by the availability of the other EAAs.

Studies of muscle protein synthesis in rats have limited relevance to human responses. Skeletal muscle comprises a much smaller percentage of the total body mass in rats as compared to humans and regulation of muscle protein synthesis differs in many respects. Thus, in their landmark book on protein metabolism Waterlow and associates concluded from available data that dietary amino acids do not stimulate muscle protein synthesis in rats [5]. While recent work challenges this assertion, the limited stimulatory effect of dietary amino acids on protein synthesis in the rat reflects the fact that under normal post-absorptive conditions there are excess endogenous amino acids available to enable an increase in protein synthesis if the activity of intracellular factors involved in the initiation of protein synthesis is stimulated. Expressed differently, muscle protein synthesis in the rat is apparently limited by the initiation process rather than the translation process. In contrast, as will be discussed below, that does not appear to be the case in humans. Another important distinction between studies investigating the effects of amino acids on muscle protein synthesis in humans and rats relates to the methodologies commonly used. The “flooding dose” technique [6] has usually been used in rat studies. This procedure involves measurement of the incorporation of an amino acid tracer into muscle protein over a very short time window, often as short as 10 min. This approach does not distinguish between a transient and a sustained stimulation of protein synthesis. Only a sustained stimulation of synthesis is relevant physiologically. Consumption of an imbalanced mixture of amino acids, such as the BCAAs, may transiently stimulate protein synthesis by utilizing endogenous stores of the other precursors of protein synthesis. However, endogenous stores of amino acids, such as those in plasma and free intracellular pools, are quite limited and may quickly become depleted. If the stimulation of protein synthesis cannot be sustained, there is little physiological significance. Consequently, the flooding dose technique commonly used to measure muscle protein synthesis in the rat produces results with uncertain relevance to human nutrition. Since BCAA dietary supplements are intended for human consumption, the focus of this short review will be research in human subjects.

The sale of BCAAs as nutritional supplements has become a multi-million dollar business. At the center of the marketing for these products is the widely-believed claim that consumption of BCAAs stimulates muscle protein synthesis, and as a result elicits an anabolic response. BCAAs may also be consumed for the purpose of improving “mental focus”, but we will not consider that application. The primary purpose in this paper to evaluate the assertion that BCAAs alone are anabolic is adequately supported either theoretically or empirically by studies in human subjects. Implicit in our assessment will be the examination of whether or not the phosphorylation state of the eukaryotic initiation factors plays a rate-controlling role in the regulation of muscle protein synthesis in humans.

### Muscle protein turnover and dietary protein intake

Muscle protein is in a constant state of turnover, meaning that new protein is continuously being produced while older proteins are being degraded. The anabolic state has no specific definition, but generally refers to the circumstance in which the rate of muscle protein synthesis exceeds the rate of muscle protein breakdown. The results in a gain of muscle mass. Conventionally the anabolic state is considered to be driven by a stimulation of muscle protein synthesis, but theoretically could also result from an inhibition of muscle protein breakdown.

The overriding metabolic goal of consuming BCAA supplements is to maximize the anabolic state. It is widely asserted that BCAAs induce an anabolic state by stimulating muscle protein synthesis. An abundant availability of all EAAs is a requisite for a significant stimulation of muscle protein synthesis [7]. Muscle protein synthesis will be limited by the lack of availability of any of the EAAs, whereas a shortage of NEAAs can be compensated for by increased de novo production of the deficient NEAAs [7]. In the post-prandial state following a meal containing protein, all of the EAA precursors required for new muscle protein synthesis can be derived from either the elevated plasma concentrations resulting from digestion of the consumed protein or from recycling from protein breakdown. In this circumstance of abundant availability of EAAs the rate of muscle protein synthesis exceeds the rate of breakdown, thereby producing an anabolic state. In the post-absorptive state the plasma EAA levels fall below the post-prandial values because amino acids are no longer being absorbed. As a result, EAAs are no longer taken up by muscle, but rather released by muscle into plasma [8]. This catabolic state of muscle protein in the post-absorptive state enables continued availability of EAAs for other tissues to maintain the rate of protein synthesis at the expense of muscle protein, which can be considered to play a role as the reservoir of EAAs for the rest of the body to draw upon.

Since EAAs cannot be produced in the body and there is a net release of EAAs from muscle, in the post-absorptive state the only source of EAA precursors for muscle protein synthesis is intracellular EAAs derived from muscle protein breakdown [8]. In addition to being reincorporated into muscle protein via synthesis, some EAAs released from muscle protein breakdown may be partially oxidized within muscle, thereby making them unavailable for reincorporation into muscle protein. EAAs released from muscle protein breakdown that are not reincorporated into muscle protein or oxidized within muscle tissue are released into plasma, whereupon they can either be taken up by other tissues as precursors for protein synthesis or irreversibly oxidized [9]. Thus, the rate of muscle protein synthesis will always be lower than the rate of muscle protein breakdown in the post-absorptive state, owing to the net flux of EAAs from protein breakdown into plasma and to oxidative pathways. Expressed differently, it is impossible for muscle protein synthesis to exceed the rate of muscle protein breakdown when the precursors are derived entirely from protein breakdown, and thus an anabolic state cannot occur in the absence of exogenous amino acid intake.

### Are BCAAs anabolic in the post-absorptive state?

#### Theoretical considerations

**Fig. 1:**
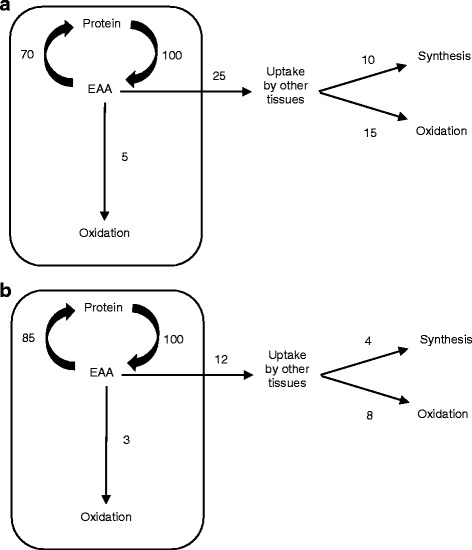
Schematic representation of the recycling of essential amino acids (EAAs) from muscle protein breakdown into muscle protein synthesis in the post-absorptive state. Arbitrary units are used for simplicity and are based on measured rates of each pathway in post-absorptive human subjects [10]. **a** Normal circumstance in the post-absorptive state. Approximately 70% of EAAs from muscle protein breakdown are recycled into protein synthesis [10]. There is a net efflux of approximately 85% of EAAs released from protein breakdown, which can either be taken up and incorporated into protein in other tissues or oxidize. About 15% of EAAs from protein breakdown are partially oxidized in muscle and unavailable for protein synthesis. The figures for outward flux and intracellular oxidation of EAAs are averages, since some EAAs, such as phenylalanine, are not oxidized at all in muscle. **b** Representation of a 50% increase in efficiency of recycling of EAAs from muscle protein breakdown into protein synthesis. In this example there would be an increase in synthesis from 70 to 80 units, or 20%. Protein synthesis can never exceed protein breakdown in the post-absorptive state, since protein breakdown is the only source of EAAs

All EAA precursors for muscle protein synthesis in the post-absorptive state are derived from muscle protein breakdown. It has been consistently reported that in normal post-absorptive humans the rate of muscle protein breakdown exceeds the rate of muscle protein synthesis by approximately 30% [10]. Consumption of BCAAs alone (i.e., without the other EAAs) can only increase muscle protein synthesis in the post-absorptive state by increasing the efficiency of recycling of EAAs from protein breakdown back into protein synthesis, as opposed to either being released in to plasma or oxidized. This is because all 9 EAAs (as well as 11 NEAAs) are required to produce muscle protein, and EAAs cannot be produced in the body. If only 3 EAAs are consumed, as is the case with consumption of BCAAs, then protein breakdown is the only source of the remaining EAAs required as precursors for muscle protein synthesis. It is therefore theoretically impossible for consumption of only BCAAs to create an anabolic state in which muscle protein synthesis exceeds muscle protein breakdown. If the generous assumption is made that BCAA consumption improves the efficiency of recycling of EAAs from muscle protein breakdown to muscle protein synthesis by 50%, then this would translate to a 15% increase in the rate of muscle protein synthesis(30% recycled in basal state X 50% improvement in recycling = 15% increase in synthesis). Further, a 50% reduction in the release of EAAs into plasma from muscle would also reduce the plasma and intracellular pools of free EAAs. Figure Fig. 1 schematically illustrates these principles. Since a 50% improvement in recycling efficiency would be about the reasonable maximal limit, this means that the maximal stimulation of muscle protein synthesis could not exceed 15%. This would correspond to an increase in the fractional synthetic rate of muscle from a basal value of about 0.050%/h in the basal state to 0.057%/h, and this difference in the fractional synthetic rate (FSR) of protein would be difficult to accurately measure [11].

#### Empirical results

BCAAs have been administered intravenously in the only studies determining the response of muscle protein metabolism in human subjects to BCAAs alone. While the infusion of BCAAs is not the conventional manner in which a dietary supplement would be consumed, intravenously infused and orally-ingested amino acids have been shown to elicit comparable effects on muscle protein synthesis in other circumstances [12]. Consequently, it is reasonable to evaluate the papers in which the response of muscle protein synthesis to the intravenous infusion of BCAAs in human subjects.

Louard et al. [13] used the forearm balance method to quantify the response to the intravenous infusion of a mixture of BCAAs for 3 h in 10 post-absorptive subjects. The forearm balance method involves the measurement of the uptake and release of individual EAAs (leucine and phenylalanine in this case) and their isotopically-labelled counterparts. Rates of disappearance (Rd) and appearance (Ra) of phenylalanine and leucine are calculated. With the assumption that the balance across the muscle of leucine and phenylalanine is representative of all EAAs, Rd. of phenylalanine is taken to be a reflection of muscle protein synthesis, since protein synthesis is the only fate of phenylalanine taken up by muscle from plasma. The Rd. of leucine cannot be interpreted with regard to protein synthesis, as leucine taken up by muscle can be oxidized as well as incorporated into protein. The 3 h infusion of BCAAs increased plasma concentrations of all 3 BCAAs four-fold, while the concentrations of other EAAs decreased [13]. Rather than being stimulated by the BCAA infusion, muscle protein synthesis decreased from 37+/− 3 to 21 +/− 2 nmol/min/100 ml leg (statistically significant, *p* < 0.05) [13]. There was no significant change in net phenylalanine balance, indicating that muscle protein breakdown was also reduced an amount similar to the reduction in muscle protein synthesis. The balance between muscle protein synthesis and breakdown remained negative, meaning that the catabolic state persisted and an anabolic state was not produced. The simultaneous decreases in muscle protein synthesis and breakdown during BCAA infusion can be described as decreased muscle protein turnover.

Similar results were obtained by the same investigators when they extended the infusion of BCAA to 16 h in 8 normal volunteers and determined if chronic elevation of BCAAs stimulated muscle protein synthesis [14]. The same forearm balance methodology was used as in the previous study to calculate muscle protein synthesis and breakdown. The 16 h infusion increase BCAA concentrations from 5 to 8 fold [14], which is as much as double the levels achieved with a normal dose of BCAAs ingested orally [15]. As in the previous study, muscle protein synthesis (as reflected by phenylalanine Rd) was reduced in the subjects receiving BCAAs as compared to saline infusion from 36 +/− 5 to 27 +/−2 nmol/min/100 ml.Muscle protein breakdown was also reduced, meaning that muscle protein turnover was reduced as well, and a catabolic state persisted.

We can conclude from these two studies that BCAA infusion not only fails to increase the rate of muscle protein synthesis in human subjects, but actually reduces the rate of muscle protein synthesis and the rate of muscle protein turnover. The catabolic state was not reversed to an anabolic state in either study. Further, a sustained reduction in the rate of muscle protein turnover would be expected to have a detrimental effect on muscle strength, even if muscle mass is maintained. Muscle protein turnover renews the muscle fibers and results in increased efficiency of contraction at the single fiber level [16], which is reflected in increased strength in vivo, independent of muscle mass [17, 18].

The failure of muscle protein synthesis to increase significantly in response to the infusion of BCAAs alone is as expected according to the theoretical considerations discussed above and illustrated in Fig. Fig. 1 with regard to the requirement for all EAAs to sustain an increase. Instead, since muscle protein breakdown decreased, the availability of EAAs also fell, which in turn actually reduced the rate of muscle protein synthesis.

#### Are the anabolic signaling factors rate-limiting in the post-absorptive state?

The claim that muscle protein synthesis is stimulated by the BCAAs stems at least in part from the observation that intracellular anabolic signaling is increased, including the activation state of key factors involved in the initiation of protein synthesis [1]. The theory that activation of intracellular anabolic signaling factors causes an increased rate of muscle protein synthesis has become entrenched in modern concepts of the regulation of muscle protein synthesis. Increased anabolic signaling in response to BCAAs has been cited as evidence of a stimulation of muscle protein synthesis, even in the absence of the measurement of muscle protein synthesis (e.g., [1]). However, activation of the anabolic signaling pathways can only coincide with increased muscle protein synthesis if there are ample EAAs to provide the necessary precursors to produce complete protein.

Dissociation of the phosphorylation state of the signaling factors and muscle protein synthesis in humans has been shown in a variety of circumstances when the availability of all of the EAAs is limited. For example, an increase in insulin concentration (for example, as a result of glucose intake) is a potent activator of the anabolic signaling pathways, but this fails to increase muscle FSR because of a deficiency of EAAs [19]. Conversely, consumption of a small amount (3 g) of EAAs stimulates muscle protein synthesis without affecting initiation factor activity e.g., Akt, S6 kinase, and 4E–BP1 [20]. A small increase in plasma concentrations of EAAs would have no effect if protein synthesis was limited by the activation state of the initiation factors. In the studies cited above in which BCAAs were infused intravenously, it is reasonable to presume that such a large increase in BCAA concentrations would have activated the signaling factors, yet muscle protein synthesis actually decreased due to lack of availability of EAAs resulting from a decrease in protein breakdown. Thus, in human subjects provision of EAAs can increase muscle protein synthesis in the absence of any change in the activation of initiation factors, and activation of the initiation factors in the absence of consumption of all of the EAAs has no effect on muscle protein synthesis. These results can only be interpreted as demonstrating that the rate-limiting control of basal muscle protein synthesis in humans is availability of all of the EAAs as opposed to anabolic signaling factor activity. This conclusion casts further doubt on the role of dietary supplement of BCAAs alone as stimulators of muscle protein synthesis.

When all evidence and theory is considered together, it is reasonable to conclude that there is no credible evidence that ingestion of a dietary supplement of BCAAs alone results in a physiologically-significant stimulation of muscle protein. In fact, available evidence indicates that BCAAs actually decrease muscle protein synthesis. All EAAs must be available in abundance for increased anabolic signaling to translate to accelerated muscle protein synthesis.

### BCAA co-ingestion with other nutrients

The focus of this review has been the response to BCAAs alone, as this is the logical intent of BCAA nutritional supplements. As in the case of consumption of BCAAs alone, there are limited studies of the co–ingestion of BCAAs with other nutrients. When BCAAs or an isonitrogenous mixture of threonine, methionine and histidine were administered to human subjects along with carbohydrate, the rate of muscle protein synthesis decreased equally in both groups, indicating no unique role of the BCAAs [21]. Similarly, consumption of a mixture of BCAAs to carbohydrate after resistance exercise did not increase the anabolic signaling factors to any greater extent than carbohydrate alone [22]. Thus, available evidence does not support the notion of a special anabolic effect of the BCAAs when given with carbohydrate.

In contrast to the lack of an interactive effect between BCAAs and carbohydrate, BCAAs may enhance the anabolic effect of a protein meal. For example, the addition of 5 g of BCAAs to a beverage containing 6.25 g whey protein increased muscle protein synthesis to a level comparable to that induced by 25 g of whey protein [23]. This result suggests that one or more of the BCAAs might be rate limiting for the stimulation of muscle protein synthesis by whey protein, or that the extra BCAAs induced a greater potential for an anabolic response of muscle to whey protein by activating the initiation factors. In either case, the response of BCAAs in conjunction with intact protein is a different issue that the effect of BCAAs alone, since the intact protein provides all of the EAAs necessary to produce an intact protein.

### Individual effects of leucine, valine and isoleucine

In this paper we have considered only the response to mixtures of BCAAs. The responses to individual BCAAs (i.e., leucine, valine or isoleucine) might differ from the combination of the three for several reasons. Evidence indicates that leucine alone may exert and anabolic response (e.g., [24]), while no such data exists for isoleucine or valine. Thus, it might be expected that leucine alone would be more effective than the combination of all of the BCAAs. However, there are two significant limitations of a dietary supplement of leucine alone. First, the same issues that limit the extent of stimulation of muscle protein synthesis by BCAAs alone regarding the availability of the other EAAs necessary for the production of intact muscle protein also limit the response to leucine alone. Second, elevation of the plasma concentration of leucine activates the metabolic pathway that oxidizes all of the BCAAs. As a result, ingestion of leucine alone results in a decrease in the plasma concentrations of both valine and isoleucine. The availability of valine and isoleucine may therefore become rate limiting for muscle protein synthesis when leucine alone is consumed. This may be why long-term outcome studies with dietary leucine supplementation have failed to yield positive results [25]. The principal rationale for a dietary supplement containing all of the BCAAs as opposed to leucine alone is to overcome the decreases in plasma concentrations of valine and isoleucine that would occur when leucine is given alone.

While a dietary supplement with all of the BCAAs will overcome the decreases in concentration resulting from consumption of leucine alone, the addition of valine and isoleucine may nonetheless limit the effectiveness of leucine alone due to competition for transport into muscle cells. The BCAAs are all actively transported into cells, including muscle cells, by the same transport system. Therefore, when provided together the BCAAs compete with each other for transport into the cells. If one of the BCAAs (e.g., leucine) is rate limiting for protein synthesis, addition of the other two BCAAs might limit the stimulation of protein synthesis because of reduced entry of leucine into the cell. The BCAAs also compete with other amino acids for transport, including phenylalanine, and this competition could affect the intramuscular availability of other EAAs. As a result of competition for transporters, it is possible that leucine alone, for example, could have a transitory stimulatory effect on muscle protein synthesis (e.g., [21]) where the BCAAs fail to elicit such response [13, 14].

## Conclusion

A physiologically-significant increase in the rate of muscle protein synthesis requires adequate availability of all amino acid precursors. The source of EAAs for muscle protein synthesis in the post-absorptive state is the free intracellular pool. Intracellular free EAAs that are available for incorporation into protein are derived from muscle protein breakdown. Under normal conditions about 70% of EAAs released by muscle protein breakdown are reincorporated into muscle protein. The efficiency of reincorporation of EAAs from protein breakdown back into muscle protein can only be increased to a limited extent. For this fundamental reason, a dietary supplement of BCAAs alone cannot support an increased rate of muscle protein synthesis. The availability of the other EAAs will rapidly become rate limiting for accelerated protein synthesis. Consistent with this perspective, the few studies in human subjects have reported decreases, rather than increases, in muscle protein synthesis after intake of BCAAs. We conclude that dietary BCAA supplements alone do not promote muscle anabolism.
